# Diagnostic Potential of a Luminex-Based Coronavirus Disease 2019 Suspension Immunoassay (COVID-19 SIA) for the Detection of Antibodies against SARS-CoV-2

**DOI:** 10.3390/v13060993

**Published:** 2021-05-26

**Authors:** Tove Hoffman, Linda Kolstad, Johanna F. Lindahl, Bo Albinsson, Anders Bergqvist, Bengt Rönnberg, Åke Lundkvist

**Affiliations:** 1 Department of Medical Biochemistry and Microbiology, Zoonosis Science Centre, Uppsala University, Husargatan 3, SE-751 23 Uppsala, Sweden; linda.kolstad@imbim.uu.se (L.K.); johanna.lindahl@imbim.uu.se (J.F.L.); bo.albinsson@imbim.uu.se (B.A.); bengt.ronnberg@gmail.com (B.R.); ake.lundkvist@imbim.uu.se (Å.L.); 2 Laboratory of Clinical Microbiology, Uppsala University Hospital, Dag Hammarskjölds väg 38, SE-752 37 Uppsala, Sweden; anders.bergqvist@medsci.uu.se; 3 Department of Medical Sciences, Uppsala University, Dag Hammarskjölds väg 38, SE-751 84 Uppsala, Sweden

**Keywords:** COVID-19, SARS-CoV-2, Coronavirus, serology, IgM, IgG

## Abstract

Due to the current, rapidly increasing Coronavirus disease 2019 (COVID-19) pandemic, efficient and highly specific diagnostic methods are needed. The receptor-binding part of the spike (S) protein, S1, has been suggested to be highly virus-specific; it does not cross-react with antibodies against other coronaviruses. Three recombinant partial S proteins of severe acute respiratory syndrome coronavirus-2 (SARS-CoV-2) expressed in mammalian or baculovirus-insect cells were evaluated as antigens in a Luminex-based suspension immunoassay (SIA). The best performing antigen (S1; amino acids 16-685) was selected and further evaluated by serum samples from 76 Swedish patients or convalescents with COVID-19 (previously PCR and/or serologically confirmed), 200 pre-COVID-19 individuals (180 blood donors and 20 infants), and 10 patients with acute Epstein-Barr virus infection. All 76 positive samples showed detectable antibodies to S1, while none of the 210 negative controls gave a false positive antibody reaction. We further compared the COVID-19 SIA with a commercially available enzyme immunoassay and a previously evaluated COVID-19 rapid antibody test. The results revealed an overall assay sensitivity of 100%, a specificity of 100% for both IgM and IgG, a quantitative ability at concentrations up to 25 BAU/mL, and a better performance as compared to the commercial assays, suggesting the COVID-19 SIA as a most valuable tool for efficient laboratory-based serology.

## 1. Introduction

In late 2019, the Coronavirus disease 2019 (COVID-19), caused by the severe acute respiratory syndrome coronavirus-2 (SARS-CoV-2), started to rapidly spread globally. On March 11 2020, it was classified as the second pandemic of the 21st century by the World Health Organization (WHO) [1]. SARS-CoV-2 belongs to the *Betacoronavirus* genus of the *Coronaviridae* family, together with SARS-CoV and the Middle East respiratory syndrome CoV (MERS-CoV), and it is the third CoV that has emerged from animals to humans within less than two decades [2]. CoVs are enveloped single-stranded RNA viruses that share structural similarities and are composed of 16 non-structural proteins and four structural proteins: the spike (S), envelope (E), membrane (M), and nucleocapsid (N) proteins. The N-terminal part of the S protein (S1) contains a receptor-binding domain (RBD) that for SARS-CoV-2 specifically recognizes the angiotensin-converting enzyme 2 (ACE2) receptor in humans [3,4,5]. The total nucleotide sequence similarity between SARS-CoV-2 and SARS-CoV is approximately 79%, and between SARS-CoV-2 and MERS-CoV it is about 50% [6].

Efficient methods for nucleic acid testing (RT-PCRs, RT-qPCRs), whole genome sequencing, virus isolation, antigen detection, and serological assays were developed within weeks or months after the initial start of the global spread of SARS-CoV-2. Although PCR-diagnosis of acute SARS-CoV-2 infections is highly efficient, specific serology is a valuable complement due to the limited time of viral RNA-positivity [7]. During the spring of 2020, several commercial serological assays such as various rapid tests and enzyme immunoassays (EIA) also became available, for a review see [8]. Previous approaches to serologic detection of infection with emerging coronaviruses, including SARS and MERS, focused on the S and N proteins, which are considered the immunodominant antigens for these viruses [9]. The S1 of MERS-CoV and SARS-CoV-2 have been proven to be highly virus-specific; i.e., non-reactive with antibodies directed to other coronaviruses [10,11].

Due to the fact that SARS-CoV-2 can (i) spread rapidly, (ii) resemble other viral and bacterial infections, (iii) cause a wide range of clinical symptoms—from asymptomatic infections to those with mild common cold symptoms or more severe symptoms, such as acute respiratory distress syndrome or multi-organ failures [12]—and (iv) result in asymptomatic carriers in up to 50% of the cases [13,14], it is of uttermost importance to develop efficient and highly specific diagnostic methods for COVID-19. We recently used the Luminex-system for the development of multiplex detection of antibodies to hantaviruses and a number of flaviviruses pathogenic to humans (e.g., dengue, Japanese encephalitis, yellow fever, Zika, and TBE viruses) [15,16,17,18]. The multiplex TBEV assay, based on whole virus antigen and the non-structural 1 (NS1) antigen, has proven most efficient for diagnosis of TBE vaccine failures [15]. Based on these earlier positive experiences of Luminex-based assays, we used the same methodology to develop and evaluate a COVID-19 assay based on the N-terminal part of the S protein (aa 16-685) of SARS-CoV-2. We aimed to investigate the presence of antibodies directed to the N-terminal part of the S protein of SARS-CoV-2 in patients and convalescents with COVID-19 and to evaluate if such an antigen alone could prove to be a useful tool for the diagnosis of the disease as well as for seroepidemiological studies.

## 2. Materials and Methods

### 2.1. Control Samples

Positive controls were collected between April and July 2020 and included serum from Swedish patients and convalescents with COVID-19 (number of days between symptom onset/PCR-positivity and serum sampling: 3–126 (median: 21.5)) confirmed positive for SARS-CoV-2 by reverse transcription real-time polymerase chain reaction (RT-qPCR) and/or by serology. In short, nucleic acid was extracted from nasopharynx samples using NucliSENS^®^ eMAG^®^ (bioMerieux, Marcy-l’Étoile, France). Presence of viral RNA was assessed by detection of the SARS-CoV-2 E and N genes using RT-qPCR, according to previously described protocols from Charité [19] and the Center for Disease Control (CDC) of the United States (US) [20]. For reverse transcription and qPCR, the TaqMan Fast Virus 1-step Master Mix (ThermoFisher Scientific, Waltham, Massachusetts, US) was used according to the manufacturer´s instructions. The reactions were performed with a sample volume of 5 μL in a total volume of 25 μL. Primer and probe concentrations for the E (E_Sarbeco_F (Forward)/_R (Reverse); E Sarbeco_P1 (Probe) and N (2019-CoV_N1-F/-R; 2019-CoV_N1-P) genes were 400 nM and 200 nM, respectively. The probes were labelled with Yakima Yellow and FAM as fluorophores, with internal ZEN and terminal 3IABkFQ as quenchers. The RT-qPCR analysis was performed on a RotorGene Q instrument (Qiagen, Hilden, Germany) with the software v. 2.3.1. The thermal cycling steps were: 50 °C for 15 minutes (min), 95 °C for 2 min, 45 cycles of 95 °C for 15 seconds (s), and 60 °C for 30 s. For the serological determination of the positive controls, the commercial assays Abbott Architect (SARS-CoV-2 specific IgG, N-based, Abbott Park, Illinois, United States), Diasorin Liaison (SARS-CoV-2 specific IgG, S1/S2-based, DiaSorin S.p.A. Saluggia, Italy), and/or the COVID-19 IgG/IgM rapid test cassette (SARS-CoV-2 specific IgG and IgM, S1-based, Zhejiang Orient Gene/Healgen) were used.

Negative controls included serum from the following: (i) infants (6–14 months old), (ii) randomly selected blood donors from the Uppsala Academic Hospital, and (iii) patients with a clinical picture of acute EBV infection/infectious mononucleosis (known to be problematic in serological assays due to polyclonal immunoglobulin activation [21]), defined as IgM-positive for Epstein-Barr virus (EBV) viral capsid antigen on the Abbott Architect instrument; collected 2014, 2018, and 2021 respectively. Infant sera were included to broaden the negative control panel, since infants are immunologically naïve if maternal antibodies are absent. Sera from patients with acute EBV infection were included to evaluate potential non-specific reactions.

### 2.2. Recombinant SARS-CoV-2 Antigens

Three proteins were evaluated as antigens by a limited panel of SARS-CoV-2 positive and negative sera: A) SARS-CoV-2 S1 (amino acids (aa) 16-685) (Sino Biological, Beijing, China); B) SARS-CoV-2 S1 + S2 extra cellular domain (ECD) (aa 16-1213) (Sino Biological); and C) SARS-CoV-2 S1 (aa 1-674) (The Native Antigen Company, Oxfordshire, United Kingdom) (Table 1). The Wuhan-Hu-1 isolate (NCBI Accession number: YP_009724390) was used for protein constructions.

### 2.3. COVID-19 SIA

The COVID-19 suspension immunoassay (SIA) was performed similarly to the earlier described more comprehensive Flavivirus suspension multiplex immunoassay (FSMIA) and tick-borne encephalitis virus (TBEV) assays [15,16,17]. Briefly, each antigen was coupled to 2.5 × 10^6^ carboxylated differentially colour-marked magnetic beads (MagPlex microspheres, Luminex Corp., Austin, Texas, US) using sulfo-N-hydroxysulfosuccinimide (sulfo-NHS) (ThermoFisher Scientific, Waltham, MA, USA) and 1-ethyl-3-[3 dimethylaminopropyl]carbodiimide hydrochloride (EDC) (Sigma Aldrich, Merck, Darmstedt, Germany), according to the manufacturer’s instructions.

For IgG determination, serum diluted 1:25 (2 μL serum and 48 μL buffer) in PBSTT (phosphate-buffered saline supplemented with 0.5% Tween 20 and Tris (50 mM)) was added to 96-well microtiter plates. Vortexed and sonicated microsphere mixture (50 μL, 25 beads/μL PBSTT) was added to each well, giving a final serum dilution of 1:50. Subsequently, the plate was incubated for 60 min in the dark at room temperature on a plate shaker (400 rpm). Microspheres were then washed with 100 μL PBS, followed by addition of 100 μL (2 μg/mL PBSTT) biotinylated protein G (Pierce Biotechnology, ThermoFisher Scientific, Waltham, MA, USA), 30 min incubation, and washing. One hundred microliters (2 μg/mL PBSTT) streptavidin–phycoerythrin (SA-PE) (Invitrogen, ThermoFisher Scientific, Waltham, MA, USA) was then added, followed by an incubation period of 15 min. Finally, the microspheres were washed once before re-suspension in 100 μL PBS and analysis of 50 μL in a Luminex MagPix instrument (Luminex Corp., Austin, TX, USA).

For IgM determination, 2 μL serum was pre-incubated with 9 μL GullSORB (Meridian Life Science, Memphis, TN, USA) and 39 μL PBSTT to remove IgG. Each well was subsequently incubated with microsphere mixture, followed by addition of 100 μL (2 μg/mL PBSTT) biotinylated anti-human IgM (Sigma Aldrich, Merck, Darmstedt, Germany) and SA-PE conjugate as described above for detection of IgG.

The assay cut-off for positivity was calculated as the average median fluorescence intensity (MFI) plus 6 standard deviations plus 10% of 200 SARS-CoV-2 antibody-negative sera.

### 2.4. Repeatability and Reproducibility

The repeatability (intra-assay/sample variation) and reproducibility (inter-assay/run variation) of the COVID-19 SIA were determined by calculating the coefficient of variability (CV), using the formula below and MFI values generated from: (i) five replicates of one sample in one run and (ii) one sample analyzed in five different runs.

%CV = (standard deviation (MFI) * 100) / mean (MFI)

### 2.5. Extended Evaluations

The sensitivity was further evaluated using the WHO international standard for anti-SARS-CoV-2 immunoglobulins (National Institute for Biological Standards and Control (NIBSC) code: 20/136) (NIBSC, Herts, UK). The effect of various antibody levels on the diagnostic performance was evaluated using the WHO international reference panel for anti-SARS-CoV-2 immunoglobulins (NIBSC code: 20/268).

### 2.6. Commercial Methods

The WANTAI EIA (Nordic BioSite, Wayne, PA, USA), based on S1 RBD and intended for detection of total SARS-CoV-2 antibodies (IgG, IgM, and IgA), and a previously evaluated rapid antibody test (Zhejiang Orient Gene Biotech Co Ltd., Huzhou, Zhejiang, China/Healgen Scientific LLC, Houston, TX, USA) (Hoffman et al., Viruses, submitted), based on S1 and intended for detection of SARS-CoV-2 IgM and IgG, were used according to the manufacturer´s instructions. Eleven patient serum samples were end-point titrated by the rapid test, the EIA, and the COVID-19 SIA.

### 2.7. Data Visualization and Statistical Analysis

Data visualization was performed using GraphPad Prism 9.0.1 (GraphPad Software, San Diego, CA, USA). Calculations of binomial confidence intervals (CI) were performed in STATA 14.2 (STATACorp Ltd., College Station, TX, USA).

## 3. Results

### 3.1. Initial Evaluation of Three SARS-CoV-2 Recombinant Antigens

Antigens (Table 1) were evaluated by a limited panel of SARS-CoV-2 positive and negative sera. Non-specific reactions were observed for antigens B and C, resulting in no further evaluations (data not shown). Antigen A (S1: aa 16-685) was further evaluated by investigating optimal antigen concentration and serum dilution. The optimal amount of conjugated antigen (2, 5, 10, and 20 μg) and final serum dilution (1:20, 1:50, 1:100) were determined as 10 μg and 1:50, respectively (data not shown).

### 3.2. Repeatability and Reproducibility

The COVID-19 SIA had an intra- and inter-assay CV of 4% and 5% for IgM and 2% and 6% for IgG, respectively (Table 2). The criterium for a successful run was determined as an inter-assay CV of 10% or less.

### 3.3. Sensitivity, Specificity, and Predictive Values of the COVID-19 SIA

**Figure 1:**
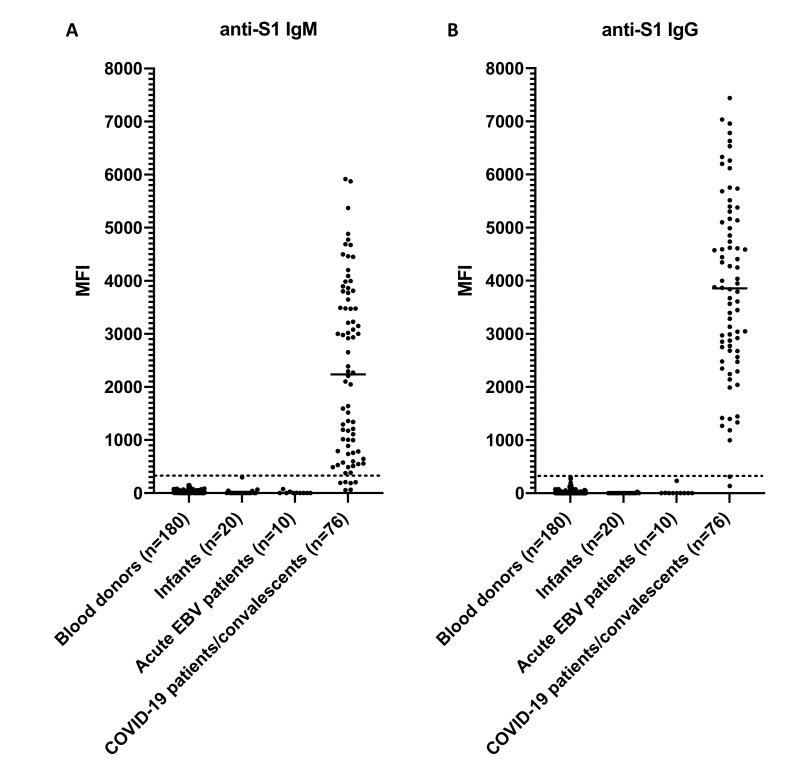
Specificity of the COVID-19 SIA. (**A**) Results for anti-S1 IgM. (**B**) Results for anti-S1 IgG. The control panel included sera from blood donors, infants, patients with acute Epstein-Barr virus (EBV) infection, and patients/convalescents with COVID-19. The dashed line indicates the assay cut-off of 300 MFI and the vertical line the median. MFI, median fluorescence intensity; Ig, immunoglobulin; COVID-19, Coronavirus disease 2019; SIA, suspension immunoassay; S1, spike subunit 1.

The assay cut-off was determined as 300 MFI. All 76 previously confirmed seropositive sera tested positive for IgM or IgG. Of those, 70 had an MFI ≥ 300 for anti-S1 IgM (range: 376–5916; median: 2520) and 75 had an MFI ≥ 300 for anti-S1 IgG (range: 313–7439; median: 3868). None of the expected seronegative samples (n = 210) collected in 2014, 2018, and 2021 tested positive for anti-S1 IgM or IgG (MFI < 300; range: 0–298). The results are summarized in Table 3 and Figure 1.

**Figure 2:**
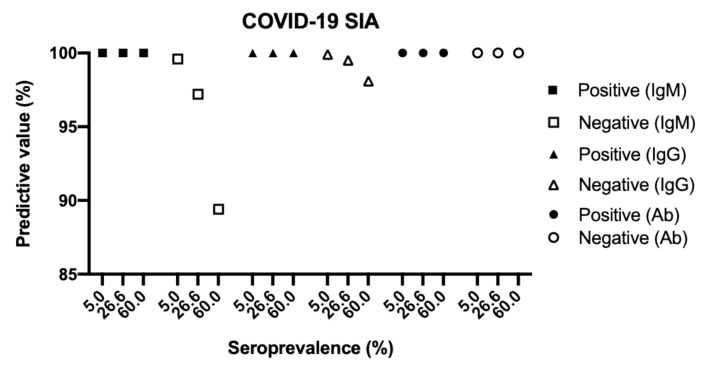
Predictive values of the COVID-19 SIA at three different seroprevalences. The predictive values were plotted as a function of seroprevalence using the values for sensitivity and specificity in Table 4 and the following formulas: Positive predictive value = (seroprevalence * sensitivity) / ((seroprevalence * sensitivity) + ((1 − seroprevalence) * (1 − specificity))); Negative predictive value = ((1 − seroprevalence) * specificity) / ((seroprevalence * (1 – sensitivity)) + ((1 – seroprevalence) * specificity)). COVID-19, Coronavirus disease 2019; SIA, suspension immunoassay; Ig, immunoglobulin, Ab, IgM + IgG.

Based on the results summarized in Table 3, the COVID-19 SIA had a sensitivity of 92.1% for IgM and 98.7% for IgG (Table 4). The assay exhibited an overall specificity of 100% for both IgM and IgG. The positive predictive values (PPV, probability of having been infected and having antibodies given a positive test result) were 100% for IgM and 100% for IgG. The negative predictive values (NPV, probability of not yet having been infected and not having antibodies given a negative test result) were 97.2% for IgM and 99.5% for IgG. PPV and NPV remained high at three different seroprevalences (5%, 26.6%, and 60%) (Figure 2).

### 3.4. Extended Evaluations

**Figure 3:**
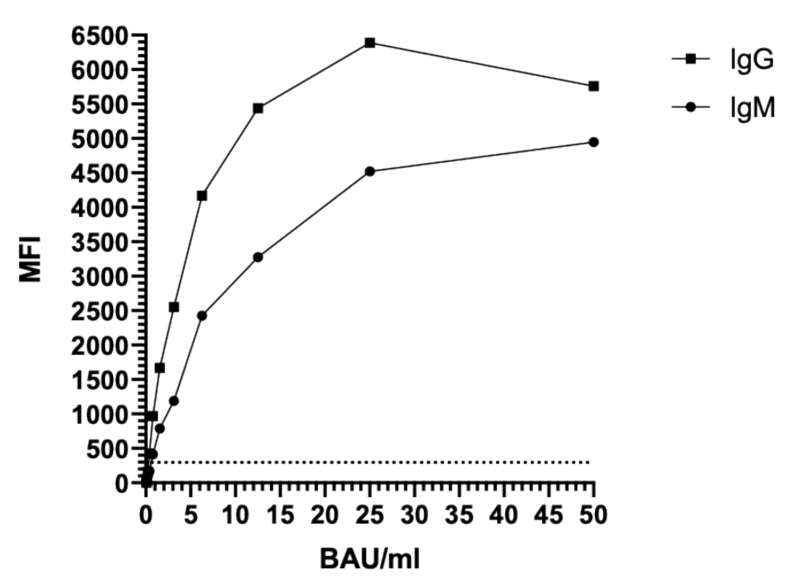
Sensitivity testing of the COVID-19 SIA, using the WHO international standard for anti-SARS-CoV-2 immunoglobulins. The dashed line indicates the assay cut-off of 300 MFI. MFI, median fluorescence intensity; BAU, binding antibody unit; Ig, immunoglobulin.

The sensitivity of the COVID-19 SIA was further evaluated using the WHO international standard for SARS-CoV-2 immunoglobulins. Results revealed a plateau for IgM and a hook (prozone) effect for IgG at concentrations higher than 25 BAU/mL (Figure 3), indicating that the COVID-19 SIA is quantitative at concentrations up to 25 BAU/mL. The limit of detection (LOD) was determined as 0.8 BAU/mL for IgM and 0.4 BAU/mL for IgG.

The effect of various antibody levels on the diagnostic performance was evaluated using the WHO international reference panel for anti-SARS-CoV-2 immunoglobulins. The results indicated coherence between the reported concentrations (BAU/mL) for the samples in the WHO international reference panel and the estimated concentrations measured by the COVID-19 SIA (Table 5).

### 3.5. Comparison to Commercially Available Assays

Eleven patient serum samples (IgM MFI range: 738–5871; IgG MFI range: 1187–6960) were end-point titrated by the COVID-19 SIA, a rapid test (Zhejiang Orient Gene/Healgen), and an EIA (WANTAI). In most cases, the SIA gave the highest end-point titers, while one sample (#1) reacted with the highest end-point titer in the EIA (Table 6).

## 4. Discussion

This study shows that the COVID-19 SIA is a sensitive, specific, robust, and relatively simple tool in the diagnostics of acute and previous SARS-CoV-2 infections. The immunoglobulins IgM and IgG are commonly used as markers for early and late infections. The COVID-19 SIA was found to have an optimal specificity for IgG detection, shown by a PPV of 100% regardless of selected seroprevalence, which indicates that the assay would be a valuable tool for studies on seroprevalences and future analyses of vaccine responses. Furthermore, the assay was found to have an overall sensitivity of 100% and a sensitivity of 92.1% and 98.7% for IgM and IgG, respectively. We used samples from COVID-19 cases obtained during disease or convalescence that were previously confirmed by PCR and/or serology as true positives. Six of the positive samples tested negative for IgM, which may be explained by the fact that they were from convalescents who no longer had detectable levels of IgM. One of the positive samples was negative for IgG, likely due to the fact that IgG had not yet developed to detectable levels.

For standardization, the WHO international standard and reference panel for anti-SARS-CoV-2 immunoglobulins were used. The COVID-19 SIA was found to have a LOD of 0.8 BAU/mL for IgM and 0.4 BAU/mL for IgG and to be quantitative at concentrations up to 25 BAU/mL (Figure 3). Furthermore, the assay was found to give reliable results at both low and high titers (Table 5). Since SARS-CoV-2 continuously evolves, it will be necessary to continue evaluations of the assay with sera from individuals infected with emerging variants of the virus to evaluate their impact on assay performance. If needed, additional S antigens will then be included in the assay. Our aim is to further develop the method into a multiplex viral assay, designed to detect antibodies against a wide array of respiratory viruses, such alpha- and beta-coronaviruses, influenza viruses, and adenovirus.

Most individuals appear to produce antibodies against both the N and the S protein of SARS-CoV-2 [22]. Several of the most commonly used laboratory serological methods detect IgG against the N protein (anti-N-IgG) [23,24]. Presence of antibodies against the N protein do not itself prove immunity. Furthermore, anti-N-IgG appear to have a shorter duration as compared to anti-S-IgG [25,26,27], which could result in an underestimation of the seroprevalence when using N-based serological assays. The COVID-19 SIA has the advantage of being based on the part of the S protein that includes the RBD, which is the structural part of SARS-CoV-2 that is involved in viral attachment to host cells and immunity by specifically recognizing the ACE2 receptor. Measurements of neutralizing antibodies are needed in vaccine studies. Future investigations should therefore include how the IgG responses measured by our COVID-19 SIA correlate to the levels of neutralizing antibodies.

In conclusion, we find the COVID-19 SIA to be a useful tool for diagnostic purposes as well as for future immunological and epidemiological studies.

## Figures and Tables

**Table 1:** Evaluated recombinant SARS-CoV-2 antigens.

Recombinant Antigen	Target	Region (aa)	Catalogue Number	Expression System	Tag	Manufactured by
(A) SARS-CoV-2	S1	16–685	40591-V08H	HEK293 cells	His	Sino Biological
(B) SARS-CoV-2	S1+S2 ECD	16–1213	40589-V08B1	Baculovirus-insect cells	His	Sino Biological
(C) SARS-CoV-2	S1	1–674	REC1806	HEK293 cells	Sheep Fc	Native Antigen

aa, amino acid; ECD, extracellular domain; SARS-CoV-2, severe acute respiratory syndrome coronavirus-2; S1, spike subunit 1; S2, spike subunit 2; ECD, extra cellular domain; HEK, human embryonic kidney; His, histidine; Fc, Fc domain of immunoglobulin.

**Table 2:** Assay variability of the COVID-19 SIA.

Assay Variation	Intra		Inter	
Isotype	IgM (MFI)	IgG (MFI)	IgM (MFI)	IgG (MFI)
	2508	1391	2584	1446
	2584	1446	2566	1442
	2688	1411	2374	1348
	2651	1432	2525	1394
	2403	1366	2319	1263
Mean	2567	1409	2474	1379
SD	115	32	120	76
%CV	4	2	5	6

COVID-19, Coronavirus disease 2019; SIA, suspension immunoassay; SD, standard deviation; CV, coefficient of variability; Ig, immunoglobulin; MFI, median fluorescence intensity.

**Table 3:** Seropositive rate of control sera against S1 protein (aa 16-685) of SARS-CoV-2.

	IgM		IgG		Total Ab (IgM + IgG)	
	n	% (95% CI)	n	% (95% CI)	n	% (95% CI)
Blood donors	0/180	0.0 (0.0–2.0) *	0/180	0.0 (0.0–2.0) *	0/180	0.0 (0.0–2.0) *
Infants	0/20	0.0 (0.0–16.8) *	0/20	0.0 (0.0–16.8) *	0/20	0.0 (0.0–16.8) *
Patients with acute EBV	0/10	0.0 (0.0–30.8) *	0/10	0.0 (0.0–30.8) *	0/10	0.0 (0.0–30.8) *
Patients/ convalescents with COVID-19	70/76	92.1 (83.6–97.0)	75/76	98.7 (92.9–99.9)	76/76	100 (95.3–100) *

S1, spike subunit 1; RBD, receptor binding domain; CI, confidence interval; Ig, immunoglobulin; Ab, antibodies; EBV, Epstein-Barr virus; * One sided 97.5% confidence interval.

**Table 4:** Evaluation results of the COVID-19 SIA.

	IgM		IgG		Total Ab (IgM + IgG)	
	n	% (95% CI)	n	% (95% CI)	n	% (95% CI)
Sensitivity	70/76	92.1 (83.6–97.0)	75/76	98.7 (92.9–99.9)	76/76	100 (93.8–100) *
Specificity	210/210	100 (98.3–100) *	210/210	100 (97.7–100) *	210/210	100 (98.3–100) *
PPV #	70/70	100 (94.9–100) *	75/75	100 (95.2–100) *	76/76	100 (95.3–100) *
NPV #	210/216	97.2 (94.1–99.0)	210/211	99.5 (97.4–100)	210/210	100 (98.3–100) *

COVID-19, Coronavirus disease 2019; SIA, suspension immunoassay; Ig, immunoglobulin; Ab, antibodies; CI, confidence interval; PPV, positive predictive value; NPV, negative predictive value; * one sided 97.5% confidence interval; # Study seropositivity = 26.6% (76/286); #, number.

**Table 5:** Titer comparison of the WHO international reference panel for anti-SARS-CoV-2 immunoglobulins.

Antibody Titer (NIBSC Code)	NIBSC BAU/mL (Anti-S1 IgG)	COVID-19 SIA MFI ^a^ (Anti-S1 IgG)	Plot ^b^ BAU/mL	COVID-19 SIA ^c^ BAU/mL
Low (20/149)	46	468	0.39	39
Low S, High N (20/144)	50	618	0.59	59
Mid (20/148)	246	2352	2.8	280
High (20/150)	766	4052	6.14	614
Negative (20/142)	ND	9 ^d^	ND	ND

^a^ Dilution: 1:100; ^b^ Estimated from Figure 3; ^c^ Estimated concentration in original sample; ^d^ Cut-off for seropositivity = 300 MFI; COVID-19, Coronavirus disease 2019; SIA, suspension immunoassay; S1, spike subunit 1; RBD, receptor binding domain; BAU, binding antibody unit, ND, not determined.

**Table 6:** Assay comparison with titer presentation of positive serum samples.

	COVID-19 SIA		Orient Gene Rapid Test *		WANTAI ELISA
Sample #	IgM	IgG	IgM	IgG	Total Ab
1	1:2500	1:2500	1:50	1:10	1:6250
2	1:500	1:100	1:10	1:10	1:250
3	1:100	1:2500	1:1	1:10	1:250
4	1:2500	≥1:12,500	1:50	1:50	1:6250
5	1:2500	1:500	1:50	1:10	1:1250
6	1:100	1:12,500	1:10	1:10	1:250
7	1:12,500	1:12,500	1:50	1:50	1:6250
8	1:100	1:2500	1:10	1:10	1:50
9	1:2500	1:12,500	1:10	1:10	1:6250
10	1:12,500	1:12,500	1:50	1:50	1:6250
11	1:62,500	1:12,500	1:50	1:50	1:6250

Ig, immunoglobulin; Ab, antibodies; COVID-19, Coronavirus disease 2019; SIA, suspension immunoassay; * Additional 1:17 dilution (5 μL serum and 80 μL buffer during analysis). # Study seropositivity = 26.6% (76/286); #, number.

## Data Availability

Data available on request.
